# Supplementary material for: Application of One-Step Reverse Transcription Droplet Digital PCR for Dengue Virus Detection and Quantification in Clinical Specimens
Source: Diagnostics (Basel). 2021 Apr 1;11(4):639. doi: 10.3390/diagnostics11040639 (PMC8066273; doi:10.3390/diagnostics11040639)
Supplement: Supplementary file 1 [file diagnostics-11-00639-s001.zip › S1_Fig.pdf]

```
                                           10270     10280     10290     10300     10310     10320
                                  ....|....|....|....|....|....|....|....|....|....|....|....|
pGem3UTRD4-M14931(10261-10620)    tgtaattaccaacaacaaacaccaaaggctattgaagtcaggccacttgtgccacggttt

                                           10330     10340     10350     10360     10370     10380
                                  ....|....|....|....|....|....|....|....|....|....|....|....|
pGem3UTRD4-M14931(10261-10620)    gagcaaaccgtgctgcctgtagctccgccaataatgggaggcgtaataatccccagggag

                                           10390     10400     10410     10420     10430     10440
                                  ....|....|....|....|....|....|....|....|....|....|....|....|
pGem3UTRD4-M14931(10261-10620)    gccatgcgccacggaagctgtacgcgtggcatattggactagcggttagaggagacccct

                                           10450     10460     10470     10480     10490     10500
                                  ....|....|....|....|....|....|....|....|....|....|....|....|
pGem3UTRD4-M14931(10261-10620)    cccatcactgacaaaacgcagcaaaagggggcccgaagccaggaggaagctgtactcctg

                                           10510     10520     10530     10540     10550     10560
                                  ....|....|....|....|....|....|....|....|....|....|....|....|
pGem3UTRD4-M14931(10261-10620)    gtggaaggactagaggttagaggagacccccccaacacaaaacagcatattgacgctggg

                                           10570     10580     10590     10600     10610     10620
                                  ....|....|....|....|....|....|....|....|....|....|....|....|
pGem3UTRD4-M14931(10261-10620)    aaagaccagagatcctgctgtctctgcaacatcaatccaggcacagagcgccgcaagatg
```

Figure S1. The sequence of the 3´UTR conserved region of DENV4

The sequence of the 3´UTR conserved region of DENV4 cloned into pGem3UTRD4-M14931 is shown.
