# Supplementary material for: Application of One-Step Reverse Transcription Droplet Digital PCR for Dengue Virus Detection and Quantification in Clinical Specimens
Source: Diagnostics (Basel). 2021 Apr 1;11(4):639. doi: 10.3390/diagnostics11040639 (PMC8066273; doi:10.3390/diagnostics11040639)
Supplement: Supplementary file 1 [file diagnostics-11-00639-s001.zip › S1_Table.pdf]

Table S1. Raw quantification results for determining lower limit of detection ($LLOD_{95}$) and lower limit of quantification (LLOQ). Lower limit of detection ($LLOD_{95}$) and lower limit of quantification (LLOQ) were determined by serial dilution of *in vitro* transcribed RNA standards. The experiment was done in triplicate with three separate runs

Expected[c] (copies)	Dilution	Anticipated[d] (log10-copies)	Number of measurement (log10-copies) [b]									Mean	SD	%CV
			1	2	3	4	5	6	7	8	9			
10^5	Undiluted	4.67	4.78	4.78	4.74	4.53	4.72	4.74	4.54	4.59	4.63	4.67	0.10	2.15
10^4	1:10	3.67	3.44	3.36	3.35	3.15	3.34	3.36	3.25	3.26	3.32	3.31	0.08	2.51
10^3	1:100	2.67	2.11	2.14	2.03	1.87	1.97	2.13	1.88	2.03	1.99	2.02	0.10	4.95
10^2	1:1000	1.67	0.62	0.91	1.03	0.48	1.26	0.88	2.01	0.58	0.58	0.93	0.48	51.53
50	1:2000	1.37	0.62	0.58	0.94	0.01	NC[a]	NC[a]	0.48	0.08	0.72	0.38	0.34	89.41
10	1:10000	0.67	NC[a]	0.45	0.48	NC[a]	NC[a]	0.20	0.26	NC[a]	0.15	0.17	0.15	87.12
5	1:20000	0.37	0.73	0.75	0.15	0.26	NC[a]	NC[a]	NC[a]	0.15	NC[a]	0.23	0.31	137.03

[a]NC = No call for positive droplets were not detected.

[b]Number of measurement (log copies/reaction) = Measured concentration by RT-ddPCR/reaction total volume of 20 µl

[c]Conc. = Estimated concentration calculated by Qubit® 2.0 fluorometer.

[d]Anticipated Concentrations = Concentrations calculated as previously described by Persson et al [26]
