# Supplementary material for: Application of One-Step Reverse Transcription Droplet Digital PCR for Dengue Virus Detection and Quantification in Clinical Specimens
Source: Diagnostics (Basel). 2021 Apr 1;11(4):639. doi: 10.3390/diagnostics11040639 (PMC8066273; doi:10.3390/diagnostics11040639)
Supplement: Supplementary file 1 [file diagnostics-11-00639-s001.zip › S2 Table.pdf]

Table S2. DENV detection results of qRT-ddPCR and qRT-PCR by serotype.

		Positive qRT-PCR	Negative qRT-PCR
DENV1 (n = 40)	Positive RT-ddPCR	38	1
	Negative RT-ddPCR	0	1
DENV2 (n = 40)	Positive RT-ddPCR	37	0
	Negative RT-ddPCR	0	3
DENV3 (n = 40)	Positive RT-ddPCR	29	7
	Negative RT-ddPCR	1	3
DENV4 (n = 36)	Positive RT-ddPCR	36	0
	Negative RT-ddPCR	0	0
