# Supplementary material for: Application of One-Step Reverse Transcription Droplet Digital PCR for Dengue Virus Detection and Quantification in Clinical Specimens
Source: Diagnostics (Basel). 2021 Apr 1;11(4):639. doi: 10.3390/diagnostics11040639 (PMC8066273; doi:10.3390/diagnostics11040639)
Supplement: Supplementary file 1 [file diagnostics-11-00639-s001.zip › S2_Fig.pdf]

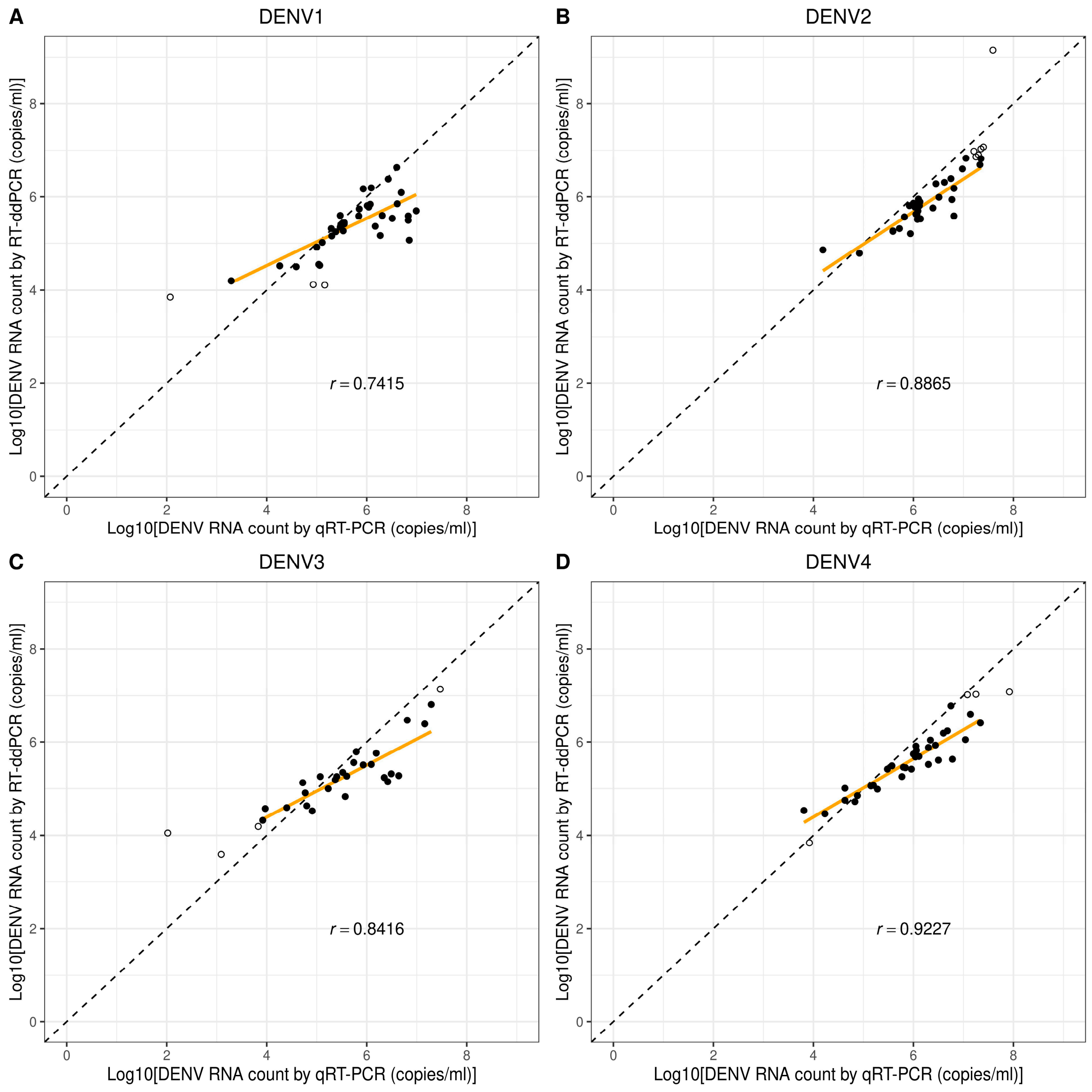

Figure S2. Comparison of quantification results from RT-ddPCR and qRT-PCR

Regression lines (orange lines) show correlations between DENV RNA from clinical samples measured by qRT-PCR and RT-ddPCR with LLOQ and ULOQ filtering. The solid dots represent measured counts within the limits of quantification. The open dots represent measured counts below LLOQ or above ULOQ which were not included in the analysis. Diagonal lines represent hypothetical trends in which results by two methods are identical. Plots are separated by serotypes: DENV1 (**A**), DENV2 (**B**), DENV3 (**C**) and DENV4 (**D**).
