# Supplementary material for: Application of One-Step Reverse Transcription Droplet Digital PCR for Dengue Virus Detection and Quantification in Clinical Specimens
Source: Diagnostics (Basel). 2021 Apr 1;11(4):639. doi: 10.3390/diagnostics11040639 (PMC8066273; doi:10.3390/diagnostics11040639)
Supplement: Supplementary file 1 [file diagnostics-11-00639-s001.zip › S3_Fig.pdf]

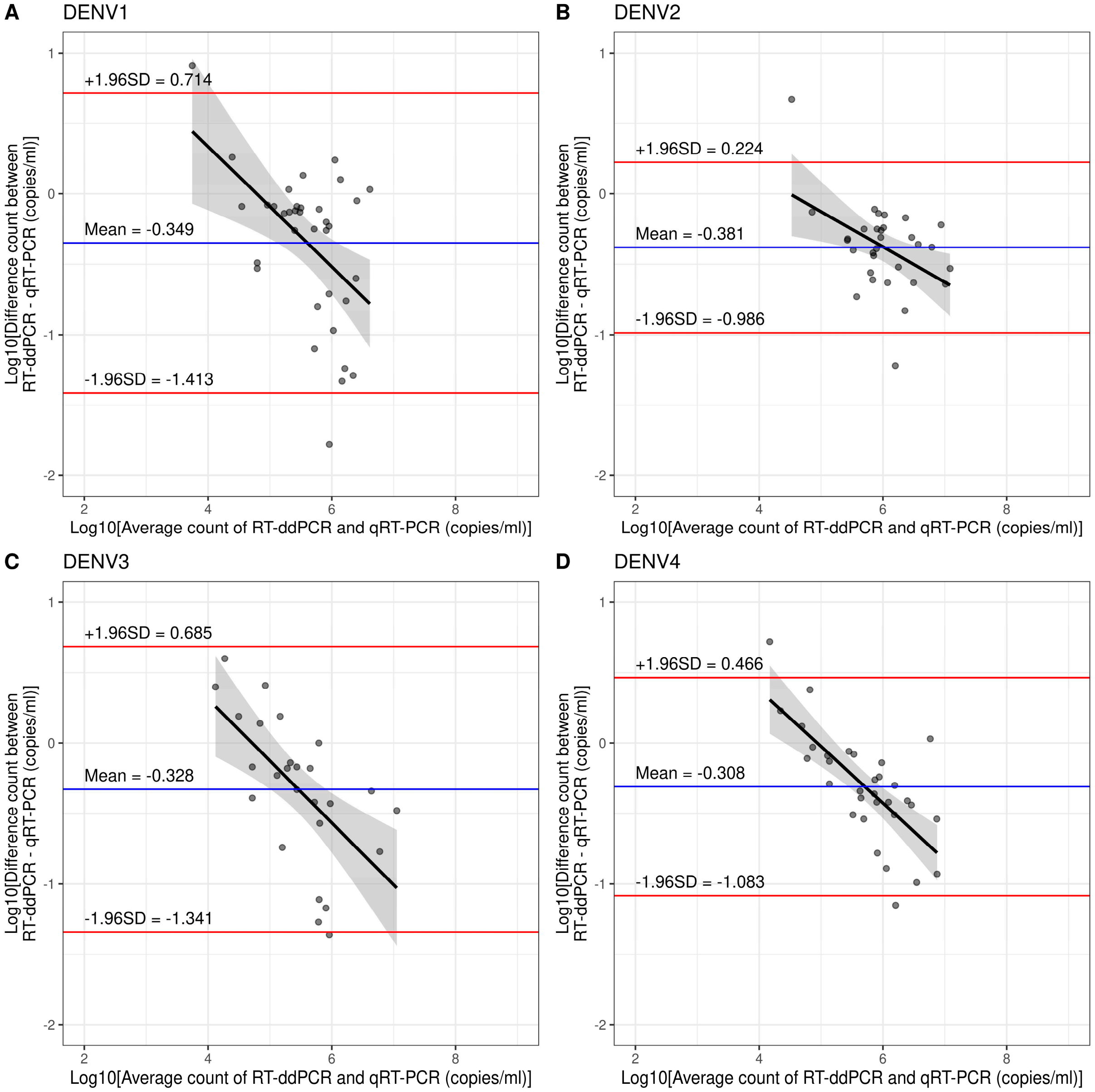

Figure S3. Bland-Altman plots for quantification agreement between qRT-PCR and RT-ddPCR

Horizontal blue lines show mean differences between the two methods (bias). Horizontal red lines show agreement limits. Linear regression lines and confidence areas show associations between measured counts and bias. Plots are separated by serotypes: DENV1 (**A**), DENV2 (**B**), DENV3 (**C**) and DENV4 (**D**).
