# Supplementary material for: Application of One-Step Reverse Transcription Droplet Digital PCR for Dengue Virus Detection and Quantification in Clinical Specimens
Source: Diagnostics (Basel). 2021 Apr 1;11(4):639. doi: 10.3390/diagnostics11040639 (PMC8066273; doi:10.3390/diagnostics11040639)
Supplement: Supplementary file 1 [file diagnostics-11-00639-s001.zip › S4_Fig.pdf]

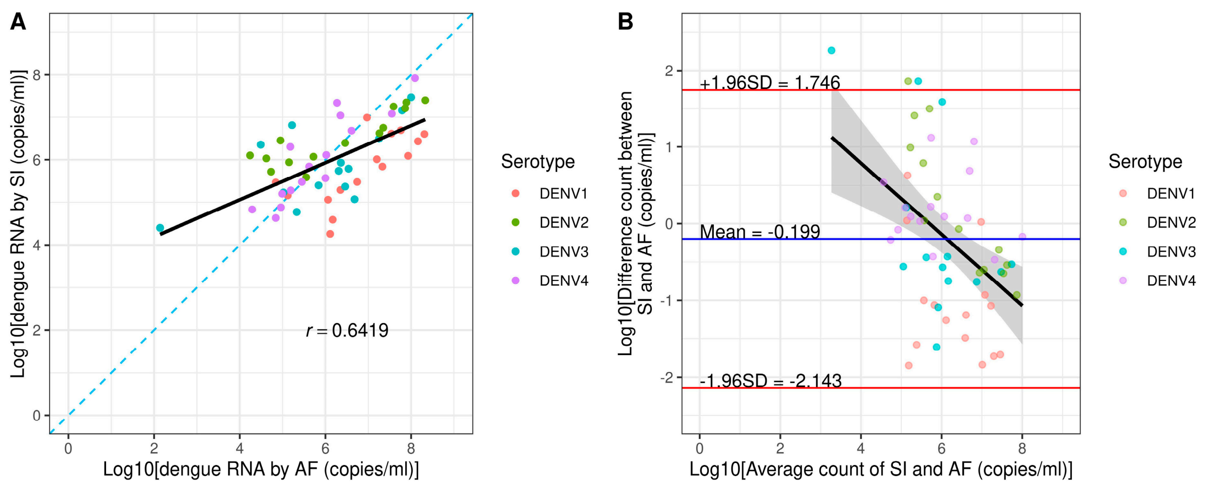

Figure S4. Interlaboratory variations of qRT-PCR

(**A**) A regression line (black line) shows the correlation between dengue RNA from clinical samples measured by qRT-PCR by two laboratories with different protocols: SI and AFRIMS (AF). A diagonal line (blue) represents a hypothetical trend where results by the two methods are identical. (**B**) A Bland-Altman plot of qRT-PCR results between AF and SI laboratories. A horizontal blue line shows mean difference between two methods (bias). Horizontal red lines show a range of agreement limits
