# Supplementary material for: Application of One-Step Reverse Transcription Droplet Digital PCR for Dengue Virus Detection and Quantification in Clinical Specimens
Source: Diagnostics (Basel). 2021 Apr 1;11(4):639. doi: 10.3390/diagnostics11040639 (PMC8066273; doi:10.3390/diagnostics11040639)
Supplement: Supplementary file 1 [file diagnostics-11-00639-s001.zip › S5_Fig.pdf]

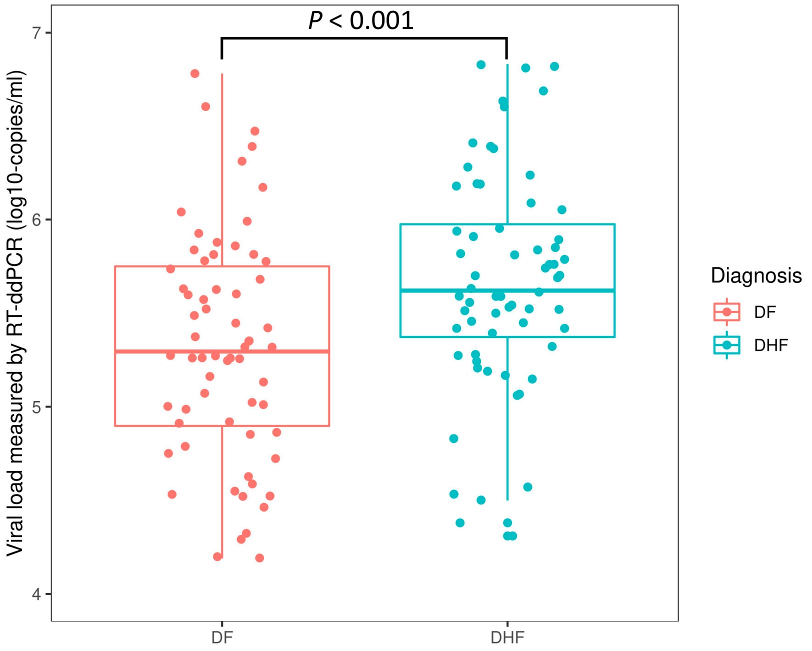

Figure S5. DENV levels grouped by disease severity

A boxplot overlaid with a scatter plot shows DENV levels grouped by disease severity. The difference of DENV levels between DF and DHF is significant ($P < 0.001$) based on a multivariate regression analysis to subtract the effect of serotype and day of illness on DENV levels.
